# Retracted: Targeting Mitochondria as Therapeutic Strategy for Metabolic Disorders

**DOI:** 10.1155/2019/6715952

**Published:** 2019-10-17

**Authors:** The Scientific World Journal

*The Scientific World Journal* has retracted the article titled “Targeting Mitochondria as Therapeutic Strategy for Metabolic Disorders” [1]. The article was found to contain a substantial amount of material without citation, from the following source:

“Mitochondrial Dysfunction in Diabetes: From Molecular Mechanisms to Functional Significance and Therapeutic Opportunities,” by William I. Sivitz and Mark A. Yorek published in Antioxidants & Redox Signaling, vol. 12, no. 4, 537–577 pages, DOI: 10.1089/ars.2009.2531, https://www.liebertpub.com/doi/full/10.1089/ars.2009.2531 [2].

## References

[1] Sorriento D., Valeria Pascale A., Finelli R. (2014). Targeting mitochondria as therapeutic strategy for metabolic disorders. *The Scientific World Journal*.

[2] Sivitz W. I., Yorek M. A. (2010). Mitochondrial dysfunction in diabetes: from molecular mechanisms to functional significance and therapeutic opportunities. *Antioxidants & Redox Signaling*.

